# Recent Biofilm Studies Open a New Door in Microbial Ecology

**DOI:** 10.1264/jsme2.ME3501rh

**Published:** 2020-03-20

**Authors:** Yoichi Kamagata

**Affiliations:** 1 Bioproduction Research Institute, National Institute of Advanced Industrial Science and Technology (AIST), Tsukuba, Ibaraki 305–8560, Japan

Biofilms comprise unique and complex microbial communities that exhibit heterologous functions in space and time (Flemming and Wingender, 2010). These microorganisms live in a self-produced matrix (biofilm) of hydrated extracellular polymeric substances (EPS), which are composed of polysaccharides, proteins, nucleic acids, or lipids. EPS account for more than 90% of biofilms, and they provide physicochemical stability by forming a three-dimensional network that functionally interconnects cells (Flemming and Wingender, 2010). Over the past two decades, the number of studies being conducted on various aspects of biofilms, including antibiotic resistance, wastewater treatment processes, quorum sensing, and the microbiota in the gastrointestinal tract, has been rapidly increasing (Fig. 1). Microbes and Environments recently published studies on biofilms.

Plastic pollution is ubiquitous and is catastrophic in marine environments. Eriksen *et al.* reported an estimate of the total number of plastic particles and their weight floating in the world’s oceans from 24 expeditions between 2007 and 2013. The estimate was a minimum of 5.25 trillion particles weighing 268,940 tons, which was markedly larger than ever expected (Eriksen *et al.*, 2014). Therefore, there is a strong and urgent need to replace extant plastics with biodegradable ones. Biodegradable plastics are classified as fossil material- and bio-based plastics (Tokiwa *et al.*, 2009). Regarding polymers, poly (3-hydroxybutyrate-*co*-3-hydroxyhexanoate) (PHBH), which is a polyhydroxyalkanoate (PHA), possesses plasticity for practical use; however, the mechanisms underlying biodegradation have not yet been elucidated. Morohoshi *et al.* investigated biofilm formation on the surface of a number of biodegradable plastics in seawater environments. They found significant amounts of biofilms on the surfaces of PHBH films (Morohoshi *et al.*, 2018a) after a 1-month incubation with seawater samples. Biofilms were comprised of diverse microbes, mainly those within Proteobacteria. While bacteria affiliated with the genus *Glaciecola* within the family Alteromonadacea dominated the surface of unbroken PHBH films, bacteria affiliated with the families Rhodobacteraceae, Rhodospirillacea, and Oceanospirillacea were dominant on the surface of partially broken PHBH films. They concluded that the biodegradation of PHBH in seawater environments may proceed through two stages: 1) biofilm formation by PHB-degrading bacteria, and 2) transition to the bacterial community that intercepts PHBH degradation products. This finding strongly suggests that initial biofilm formation on the PHBH film surface is critical for determining the subsequent stage of biodegradation. Another important finding in this study was that other bioplastics, namely, poly (lactic acid) (PLA), polybutylene succinate (PBS), poly (butylene succinate-*co*-butylene adipate) (PBSA), and poly (butylene adipate-*co*-terephthalate) (PBAT), did not exhibit any changes on their surfaces, indicating that even though they are biodegradable under certain conditions, they persist in marine environments, at least for the 1-month incubation. Besides PHBH, polyl (ε-caprolactone) (PCL) was the only polymer that showed biofilm formation and was partially degraded.

Morohoshi *et al.* also investigated the fate of PHBH in freshwater environments in a similar manner. A high throughput sequencing analysis revealed that the biofilms formed on the surfaces of PHBH and PCL films were dominated by bacteria within the genera *Acidovorax*, *Undibacterium*, *Pelomonas*, and *Ideonella*, all of which are within the order Burkholderiales (Morohoshi *et al.*, 2018b). *Acidovorax* and *Undibacterium* were isolated from the biofilm as PHBH degraders. In contrast to seawater experiments (Morohoshi *et al.*, 2018a), they found biofilm formation on the PBS, PBSA, and PBAT films (but not on PLA). Although the degradation of these polymers was not observed, these findings strongly indicate that bioplastics are more easily deteriorated in freshwater environments than in seawater environments. Therefore, the biodegradation of “bioplastics” in marine environments remains challenging.

Biofilms in wastewater treatment processes are crucial not only for treatment efficiency, but also the structural stability of microcosms. In aerobic wastewater treatments, protozoa play crucial roles in many aspects. Inaba *et al.* investigated eukaryotic microbial communities in membrane-attached biofilms in membrane bioreactors treating piggery wastewater (Inaba *et al.*, 2018). Protozoa including the phylum Ciliophora, which prey on bacterial cells, were abundant under stable conditions when membrane clogging was suppressed. Ciliates are phagotrophic protists that prey on bacterial cells, and, thus, predation by ciliates potentially inhibits membrane fouling caused by thick biofilm formation. Moreover, metazoan-like protists within the phyla Rotifera were also present, which are eukaryotes preying on protozoa that dominate the biofilm when membrane fouling progresses.

The first scientific description of hot spring biofilms (or biomats) in Japan dates back to the late 19th century (Miyoshi, 1887). Microbial communities in hot spring biofilms have attracted attention since the 1980s because microbes that thrive in extreme environments are considered to be more ancestral and closer to a primordial life than the majority of microbes under mesophilic environments. Furthermore, hydrothermal systems contain diverse geochemical conditions that indicate how an ecosystem ranging from chemolithotrophy to chemoorganotrohy functions. Ward *et al.* attempted to characterize the biofilm community structure at Jinta hot spring on Shikinejima Island (Ward *et al.*, 2019). At the hot spring, an intertidal, anoxic, iron-rich hot spring mixes with an oxygenated atmosphere and seawater over short spatial scales, creating various chemical potentials and redox pairs over a distance of ~10 m. They found that microbial communities significantly changed as temperatures and dissolved iron concentrations decreased and dissolved oxygen increased. Biomass was more limited near the spring source than downstream, and primary productivity appeared to be fueled by the oxidation of ferrous iron and molecular hydrogen by members of Zetaproteobacteria and Aquificae. Oxygenic Cyanobacteria were dominant downstream. These findings also revealed that several novel lineages within Bacteria were present, including previously uncharacterized members of the phyla Chloroflexi and Calditrichaeota.

Chloroflexi are ubiquitously distributed in natural environments. They are one of the dominant groups in hot springs. One of the earliest isolates in the phylum Chloroflexi is *Chloroflexus aggregans* (Pierson and Castenholz, 1974). *C. aggregans* is a thermophilic filamentous anoxygenic phototroph that is frequently found in microbial mats in natural hot springs. In Nakabusa hot spring, *Chloroflexus* dominates the biomat at 65°C and is present together with phototrophic cyanobacteria to possibly provide them with an organic substrate. However, it sometimes thrives as the dominant phototroph in microbial mats without cyanobacteria, strongly suggesting that *C. aggregans* has the ability to grow photoautotrophically. However, photoautotrophy had never been observed in any cultured strains of *C. aggregans*. Kanno *et al.* isolated a photoautotrophic strain from *C. aggregans*-dominated microbial mats in Nakabusa hot spring using inorganic medium under illumination (Kanno *et al.*, 2019). A new strain designated as ACA-12 was phylogenetically close to known *C. aggregans* strains. ACA-12 showed sulfide consumption along with autotrophic growth under anaerobic light conditions. The deposited elemental sulfur particles indicated the occurrence of sulfide oxidation.

Regarding Nakabusa hot spring biofilms, extensive studies have been conducted since the 1990s. Yamamoto *et al.* found that sausage-shaped microbes dominated the white filament/bundle dubbed sulfur-turf (Yamamoto *et al.*, 1998), which have not yet been cultured, but are currently recognized as bacteria within the phylum Aquificae. The biomat downstream has a layered structure from phototrophy to organotrophy. Martinez *et al.* investigated the vertical distribution and diversity of phototrophic microbes in a biomat at Nakabusa hot spring (Martinez *et al.*, 2019). They analyzed the 16S rRNA gene amplicon sequences of the biomat separated into five depth horizons, and correlated them with O_2_ concentrations and spectral scalar irradiance. A stable core community with various phototrophic organisms dominated by the filamentous anoxygenic phototrophs, *Roseiflexus castenholzii* and *C. aggregans* was identified together with the spectral signatures of bacteriochlorophyll (BChl) *a* and *c* absorption in all mat layers. In the upper mat layers, a large abundance of cyanobacteria (*Thermosynechococcus* sp.) associated with chlorophyll *a* and phycobiliprotein near the surface in a zone of high O_2_ concentrations during the day. Deeper mat layers were dominated by uncultured chemotrophic Chlorobi, which showed increasing abundance with depth (low O_2_) in these layers, indicating that they drive anaerobic metabolism.

Among the methane fermentation processes for wastewater treatment, an upflow anaerobic sludge blanket (UASB) reactor is the most efficient because microorganisms are aggregated and retained in the system as granular biofilms (van Lier *et al.*, 2015). In this process, many microbes contribute to granular biofilm formation; however, the underlying molecular mechanisms remain largely unknown. Methanogens play a crucial role in the final step of the degradation of organic matter in anaerobic processes, including UASB reactors. Recent studies have focused on methanogens, not only in terms of their methanogenic activity, but also their contribution to biofilm formation and syntrophic interactions with electrogenic bacteria. Sumikawa *et al.* attempted to identify the genes responsible for aggregation in *Methanothermobacter* sp. CaT2, which is a thermophilic hydrogenotrophic methanogen commonly found in methane-fermenting processes (Sumikawa *et al.*, 2019). A physiological analysis of the strain suggested that its cell surface sugar layer was crucial for aggregation. They isolated the aggregation-defective mutant CLA160 and compared the genome with its parental strain, and found that CLA160 had a nonsense mutation in the gene for a hypothetical membrane protein with a large extracellular domain. Together with the proteinase K treatment experiment, they concluded that the extracellular protein that has repeated sequences with a unique structure plays a key role in the aggregation of the methanogen.

In contrast to heterotrophic bacteria, methanogens are archaea characterized by their metabolic capacity. *Methanothrix* (formerly *Methanosaeta*) utilizes acetate as an energy and carbon source, but not other compounds, such as H_2_/CO_2_ and methanol/methylamines, which are common substrates for most methanogens. Despite its idiosyncratic traits, *Methanothrix* dominates anaerobic methane-fermenting consortia and plays a critical role in acetate consumption and biofilm formation due to its filamentous morphotype. *Methanothrix* has long been considered to be independent of other microbes; it simply metabolizes acetate, a low energy compound under anaerobic conditions. However, recent findings have changed our canonical view of *Methanothirx*. Kato *et al.* found that supplementation with conductive magnetite particles promoted methanogenic acetate degradation by microbial communities enriched from the production water of a high-temperature petroleum reservoir (Kato *et al.*, 2019). This study revealed that *Petrothermobacter* spp., known as thermophilic Fe(III) reducers, predominated in magnetite-supplemented enrichment, whereas other types of Fe(III) reducers, such as *Thermincola* spp. and *Thermotoga* spp., were dominant under ferrihydrite-reducing conditions. These findings suggest that magnetite induced interspecies electron transfer via electric currents through conductive particles between *Petrothermobacter* spp. and *Methanothrix*. This is the first evidence for electric syntrophy in high-temperature subsurface environments.

*Alcanivorax borkumensis* is a ubiquitous marine-dwelling bacterium that utilizes alkanes as a sole carbon source, and, thus, its biological potential as an agent of bioremediation is expected. This bacterium attaches at the oil-water interface forming interfacial communities or biofilms, which appear to mediate the consumption of spill oil (Schneiker *et al.*, 2006). Many bacteria transition between two states: planktonic and biofilms. A more detailed understanding of the planktonic-to-biofilm switch in *A. borkumensis* will provide important information on how this microbe regulates its hydrocarnonoclastic capacity depending upon the state of cell proliferation. Prasad *et al.* observed two phenotypes in the *A. borkumensis* SK2 type strain: rough (R) and smooth (S) types. The S type exhibited lower motility and higher polysaccharide production than the R type. Full genome sequencing revealed a mutation in the S type involved in cyclic dimeric guanine monophosphate (c-di-GMP) production (Prasad *et al.*, 2019). They also added hexadecane (as a model oil substrate) to observe changes in gene expression from those with pyruvate as a conventional substrate. When cultured on hexadecane, the R type exhibited marked increases in *algA* (involved in alginate synthesis) activity and a simultaneous decrease in *pilB* (involved in pilus fiber assembly) activity. Under the same conditions, they observed similar, but markedly smaller changes in the S type. These findings imply that the R type modulates its responses to different growth substrates, while the S type appears to lack this ability. Collectively, these findings clearly indicate that biofilm formation, motility, and responses to hydrocarbon are regulated by c-di-GMP levels.

Lawrence *et al.* found nickel sorption and concentrations within the exopolymeric matrix produced by communities from freshwater environments, suggesting applications to the removal of toxic elements (Lawrence *et al.*, 2019). Tanaka *et al.* developed a completely new approach to isolate microorganisms designated as “the duckweed-cocultivation method” (Tanaka *et al.*, 2018). This method is based on the finding that the duckweed rhizosphere harbors microorganisms that form biofilms, indicating that the conditions provided by the root system of duckweed are favorable for some aquatic microorganisms to grow and settle (Yamaga *et al.*, 2010). They prepared aseptic duckweeds as a recruiter plant, inoculated them with microbial communities from other plant roots, and grew them for 4‍ ‍weeks. Every week, the plant was taken and their roots were used as an inoculum for further isolation. They successfully isolated a wide variety of novel microbes, including two strains within the rarely cultivated phylum, Armatimonadetes. Many approaches including this method of co-cultivation will lead us to a new era of cultivation for as-yet cultured microbes (Tamaki, 2019). Biofilm studies are now expanding and further contributions to bioscience and technology are expected.

## Figures and Tables

**Fig. 1.**
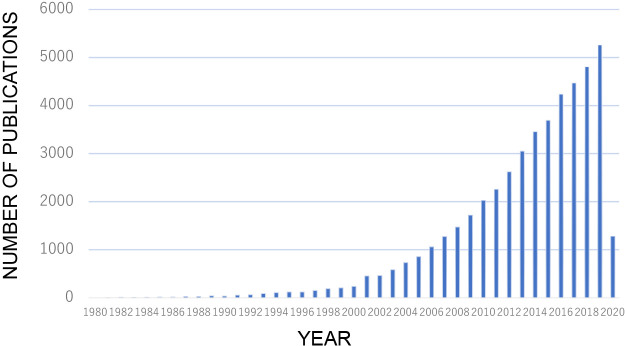
Biofilm studies over the last 40 years. Publications pertaining to biofilm are extracted from PubMed Central.
